# A significant *p* value is not equivalent to the superiority of one test index over another

**DOI:** 10.1186/s13054-019-2651-y

**Published:** 2019-11-15

**Authors:** Qiaoying Ji, Weimin Li

**Affiliations:** 1 Department of Respiratory, Affiliated Dongyang Hospital of Wenzhou Medical University, Dongyang, Zhejiang People’s Republic of China; 2 Department of Cardiology, Affiliated Dongyang Hospital of Wenzhou Medical University, Dongyang, Zhejiang People’s Republic of China

Dear Editor,

We read with great interest the study by Kazune and colleagues [1], reporting that higher mottling scores are associated with lower microcirculatory oxygen saturation. However, the authors inappropriately concluded that microcirculatory oxygen saturation was more specific than the mottling score in predicting 28-day mortality. This conclusion was based on a multivariable regression model that microcirculatory oxygen saturation was significantly associated with 28-day mortality (*p* = 0.008), but mottling score was not associated with mortality after adjustment (*p* = 0.61, 0.11, and 0.29 for the mottling scores of 1, 2 and 3, respectively, as compared with the 0 score). We have to point out that the *p* values in a multivariable logistic regression model cannot be interpreted as the strength of association, and the diagnostic performance cannot be compared with the *p* values in a multivariable regression model. The global assessment of the diagnostic performance of an index is the area under the receiver operating characteristic curve (AUROC) or concordance index. Statistical inference for the equivalence of two AUROCs can be implemented by using the Delong’s method [2]. The sensitivity and specificity may change by varying the cutoff values for determining the event versus non-event; thus, without specifying the cutoff values for the mottling score and microcirculatory oxygen saturation, it does not make sense to compare their sensitivities.

Furthermore, the authors did not make assumption on the causal relationship between these variables and just throw all of them into a multivariable regression model. Distinguishing between mediators from confounders is important. For example, the microcirculatory oxygen saturation may be a direct cause of vascular injury as measured by elevated biomarkers such as intracellular adhesion molecule-1 and vascular cell adhesion molecule-1, and have indirect effect on mortality. Such an association can be better analyzed by using the causal mediation analysis [3]. Another important issue we must point out is the influence of vasopressors on the mottling score. Pathophysiologically, vasopressors can influence the microcirculation in the skin, as well as the microcirculatory oxygen saturation as measured in the study [4]. In the framework of causal inference [5], the vasopressor use can be an important confounder because it has direct causal effect on mottling score, microcirculatory oxygen saturation, and the outcome. Such a confounder can be handled in a multivariable model, or in the counterfactual framework. However, the authors failed to do so.

## Data Availability

No data for the work.
