# Supplementary material for: A significant p value is not equivalent to the superiority of one test index over another
Source: Crit Care. 2019 Nov 15;23:359. doi: 10.1186/s13054-019-2651-y (PMC6857245; doi:10.1186/s13054-019-2651-y)
Supplement: Related texts

# Reply: Authors’ response

**Authors:** S. Kazune, A. Caica, K. Volceka, A. Grabovskis

We thank Drs Ji and Li for their interest in our article [1]. Drs Ji and Li question our conclusions regarding the accuracy of microcirculatory oxygen saturation and mottling score in predicting 28-day mortality. We would like to point out that we used *C*-statistic for assessment of diagnostic performance of microcirculatory oxygen saturation and mottling score, as suggested by Drs Ji and Li in their letter. The *C*-statistic values of univariate prediction models using microcirculatory oxygen saturation and mottling score were 0.76 and 0.79, respectively, and are both clearly stated in the results section. In the results section of our article, we also stated the cutoff values of both tests (score of 2 or more for mottling and 26% for microcirculatory oxygen saturation). Our conclusions regarding the accuracy of microcirculatory oxygen saturation and mottling score in predicting 28-day mortality are based on the above data rather than *p* values in the multivariable logistic regression model.

Another point raised by Drs Ji and Li regarding our study is the selection of independent variables for inclusion into the multivariable regression model. Although explanatory models that consider causal relationships between variables are used in etiological research, there are other purposes for the use of such models [6]. In our study, we built a descriptive model to capture the association between 28-day survival, biochemical markers of endothelial dysfunction, and skin microcirculatory hypoperfusion resulting from endothelial dysfunction.

We would also like to disagree with the objections of Drs Ji and Li regarding vasopressors as an important confounder in predicting mortality when using markers of tissue hypoperfusion such as mottling score, as this contradicts previous findings that mottling score is predictive of death irrespective of vasopressor dose [7].
